# A review on surgical treatment options in gliomas

**DOI:** 10.3389/fonc.2023.1088484

**Published:** 2023-03-16

**Authors:** Zhongxi Yang, Chen Zhao, Shan Zong, Jianmin Piao, Yuhao Zhao, Xuan Chen

**Affiliations:** ^1^ Department of Neurosurgery, The First Hospital of Jilin University, Jilin, China; ^2^ Department of Gynecology Oncology, The First Hospital of Jilin University, Jilin, China

**Keywords:** review, intraoperative techniques, extent of resection (EOR), gliomas, imaging technologies, intraoperative stimulation mapping, awake craniotomy, augmented reality high-definition fiber tractography and fluorescein

## Abstract

Gliomas are one of the most common primary central nervous system tumors, and surgical treatment remains the principal role in the management of any grade of gliomas. In this study, based on the introduction of gliomas, we review the novel surgical techniques and technologies in support of the extent of resection to achieve long-term disease control and summarize the findings on how to keep the balance between cytoreduction and neurological morbidity from a list of literature searched. With modern neurosurgical techniques, gliomas resection can be safely performed with low morbidity and extraordinary long-term functional outcomes.

## Introduction

Gliomas stem from glial cells of the central nervous system (CNS), and they are the most common primary CNS tumors. According to the 2021 World Health Organization (WHO) classification of CNS tumors, gliomas are classified as low-grade gliomas (LGGs; WHO grades I or II) and high-grade gliomas (HGGs; WHO grades III or IV). Supratentorial gliomas account for approximately 30% of all adult primary intracranial tumors, and more than 50% of these are high-grade gliomas (HGGs) (1). Several clinical features (both pathological and non-pathological) determine the grade, with pathological features including nuclear atypia, mitotic activity, vascular proliferation, necrosis, and so on, and non-pathological ones including clinical course and treatment outcome (2). Glioma is more common in whites and blacks, and the incidence of it in men is 1.5 times that in women (3).

China is one of the countries with the largest prevalence and death rates of CNS tumors (4). Overall incidence rates with adjusted age for all gliomas range from 4.67 to 5.73 per 100,000 persons (5, 6). The median overall survival (OS) times were 78.1, 37.6, and 14.4 months for low-grade gliomas, anaplastic gliomas, and glioblastomas, respectively (7). Most cases of gliomas are of sporadic onset, although some are related to Mendelian disorders such as tuberous sclerosis, neurofibromatosis type 1 or type 2, and so on (8). There are several factors that influence prognosis, including the Karnofsky Performance Status Scale at diagnosis, histology, and molecular markers. Age and tumor histology have been identified as primary predictors of patient prognosis (9, 10). Irradiated brain or scalp in the past may increase the risk of developing gliomas (11, 12).

Gliomas have variable presenting symptoms depending on tumor size and location. A systemic review summarizes the symptom prevalence and concludes several symptoms, of which the most prevalent ones are seizures, cognitive deficits, drowsiness, and dysphagia during different phases (13). In these tumors, seizures are the most common presenting symptom since they tend to be highly epileptogenic. It is common in low- and high-grade gliomas. The risk of seizures varies between 60% and 100% among low-grade gliomas and between 40% and 60% among glioblastomas (14). Seizure control and decreasing neurocognitive deficits are the two main purposes for some of the patients diagnosed with gliomas, which they always pursue.

The principle of surgical treatment of gliomas is necessary to reduce the mass effect caused by the tumors, maximize the extent of resection, and in the meantime reduce the damage to the surrounding tissue structure, especially the gliomas located in the eloquent area, to protect neurological function. The treatment procedures are different depending on the grade of the gliomas. We will introduce LGGs, HGGs, and recurrent gliomas separately.

## Low-grade gliomas

LGGs account for up to 15% of all brain tumors in adults (15). Some volumetric studies support the idea of “extent of resection” (EOR) to improve survival in patients with LGGs. These ones illustrated mean survival time variation (61.1 to 90.5 months) with maximal resection (10, 16, 17). Low-grade gliomas are the most common and uniformly fatal disease in young adults (mean age 41 years), with survival averaging approximately 7 years (18). EOR is a statistically significant predictor of overall survival. The data from these studies emphasize the importance of achieving a complete resection, cannot be overstated. LGGs have a diverse anatomical, histopathological, and molecular profile, which reflect the clinical outcome (19). In addition to affecting overall survival in LGG patients, the EOR also influences the malignant transformation rate and seizure-free status (20). A retrospective study including 153 glioma patients followed by “watch and wait” and early surgical resection, respectively, demonstrated that patients with LGGs who underwent early surgery had a higher chance of survival, which suggested that the timing of resection was crucial (21).

## High-grade gliomas

For patients with primary HGGs such as glioblastoma, retrospective analysis from a randomized trial has concluded that survival and progression-free survival are highly influenced by the extent of tumor resection; the fact that incomplete resections result in more rapid neurological deterioration also attests to the importance of complete resections on progression-free survival (22–24).

One retrospective study on the comparison of “gross total” and “subtotal” resection data demonstrated that the gross total section of HGGs had a greater survival rate for 1 year follow-up, which decreased to 19% at 2 years, than subtotal resection (25). Some MRI scans of these patients diagnosed as HGGs always show a noncontrast-enhancing surrounding abnormality, so the study of the resection rate on the abnormality’s surroundings remains unclear. Li reported the results that the group that underwent gross total resection of ≥53.21% of the surrounding FLAIR abnormality beyond the 100% contrast-enhancing resection was associated with a significant prolongation of survival compared with that following less extensive resection (p <0.001) (26).

In the discussion of the extent and rate of resection, Sanai reported that for glioblastoma multiforme (GBM), aggressive EOR equated to improvement in overall survival, even at the highest levels of resection. A significant survival advantage was seen with as little as 78% EOR, and stepwise improvement in survival was evident even in the 95%–100% EOR range (27).

## Recurrent gliomas

No matter which classification the recurrent gliomas (LGGs or HGGs) are, many patients accept repeat resection as a common treatment option to pursue a good quality of life (28). A significant benefit had been seen in 52 patients with reoperated LGGs; the principle of undergoing reoperation demonstrated an overall 10-year survival rate of 57%; variable prognostic factors such as the use of upfront radiation and pathology at recurrence influenced the overall survival rate (29). A large study on recurrent HGGs reported to date that the median overall survival duration was 19 months with a median progression-free survival following re-resection of 5.2 months, suggesting that the survival benefit of microsurgical resection did not diminish despite biological progression (30). At present, the clinical benefit of reoperation for both recurrent LGGs and HGGs demonstrates that the extent of resection can be the strongest predictor of overall survival in each individual patient.

Advances in surgical techniques and neurosurgical tools make neuro-oncology practice easier, meanwhile improving favorable outcomes and maximizing cytoreduction for patients with gliomas. Advanced neurosurgical imaging technologies, including intraoperative neuronavigation (31, 32), DTI tractography (33), intraoperative MRI (IoMRI) (34, 35), intraoperative ultrasonography (IoUS) (36, 37), fluorescence-guided surgery (38, 39), intraoperative stimulation mapping (IoSM), the awake craniotomy (AC) approach (40, 41), augmented reality high-definition fiber tractography and fluorescein (AR HDFT-F) (42, 43), intraoperative hand-held microscopy, and intraoperative mutational analysis have improved the complete radiographic resection rate of gliomas.

## Neuronavigation systems

Neuronavigation systems have been widely used in the operative management of gliomas and offer lots of advantages to surgeons. Preoperatively precise planning of the craniotomy in real time and the identification of small intracranial lesions are some of the principal benefits. In addition to the basic settings, it is also now possible to add functional MRI (fMRI) and DTI tractography information as an overlay available to the surgeon intraoperatively. fMRI does not rely on the use of radiation; it can produce images with high spatial resolution by the millimeter and poor temporal resolution because of a 5-second lag between initial neural activity and image; it can capture a clear picture of brain activity picture only if the patient stays still but not moment-to-moment brain activity. Neuronavigation can generate relationships between mass lesions and functional areas. A randomized controlled trial on the study of the effectiveness of neuronavigation demonstrated that the mean amount of residual tumor tissue was 13.8% for surgery involving neuronavigation compared with 28.9% for standard surgery, although there was no rationale for the use of neuronavigation to improve the extent of tumor resection because of the size or location of the lesion (33).

## DTI tractography

DTI is utilized to plan major fibro-parcel pathways in 3D and keep away from significant white-matter packages. The imagined white-matter groups are integrated into a 3D model to limit the level of careful dreariness brought about by disturbance of significant white-matter packs.

Nevertheless, some perioperative trials about the effect of DTI neuronavigation on reducing morbidity did not show clear evidence because of tumor infiltration and edema (31, 44, 45). Given the insufficient approximation of functional sites, DTI itself cannot be used as a tool for surgical decision-making. Diffusion-weighted MRI as a novel technique is being used to overcome the previous issue of accurately mapping peritumoral edema tissue (46).

## Intraoperative MRI

Intraoperative MRI (IoMRI) changes the way we deal with gliomas. IoMRI not only helps us to solve the problem of brain shift but also assists the neurosurgeon to highlight the tumor remnants and reach a higher EOR. In one prospective study including 100 adult patients operated on for gliomas using IoMRI with neuronavigation, Leroy reported that the median EOR was 100% whatever the type of glioma and location. It was only in the insula area that residue levels were higher. There was no difference between LGGs and HGGs in the median KPS at different follow-up times after surgery. “Staged volume” surgery was also introduced by him to ensure a high level of security for the surgeon and low morbidity for the patients (47). Several other nonrandomized studies also indicate that IoMRI can increase the resection rate of LGGs and HGGs, preserve neurofunction, and prolong patient survival (48–50).

## Intraoperative ultrasound

Intraoperative ultrasound (IoUS) is an affordable tool that can be easily incorporated into existing infrastructure and operative workflows. IoUS has dramatically evolved with well-integrated navigation tools and improvements in image quality compared with the previous artifact-prone image quality of ultrasound (51). However, it is difficult to detect residuals below 1 cm in diameter when using IoUS (52), and the cone-shaped field of view can sometimes make it hard to see the lesion.

## Fluorescence-guided surgery

Recently, intraoperative fluorescence surgery using 5-aminolevulinic acid (5-ALA) has been widely adopted (22, 53). 5-ALA as a prodrug to heme can be converted to protoporphyrin IX (PpIX), and PpIX can be accumulated preferentially in tumor cells and epithelial tissues when 5-ALA is administered through the intact blood–brain barrier (BBB) (53, 54). The properties of PpIX, which emits fluorescence of red-violet light, have been used for the detection of tumor tissues during glioma resection (38, 39).

The sensitivity of 5-ALA to gliomas has been demonstrated up to 95% in one study as a growing technology (55). Specificity for 5-ALA in predicting malignant tissues has a wide degree of variance, and in most studies it can be above 70% (56–58). The visible fluorescence varies depending on the grade of glioma, which is 95.4% in glioblastomas compared with 24.1%–26.3% in grade I and II gliomas (59). Alessandro reported the resection outcome guided by 5-ALA fluorescence: overall gross total resection of >98% was achieved in 93% of HGG patients, and the boundaries of fluorescent tissue exceeded those of tumoral tissue by neuronavigation in 43% of the patients (60).

Intraoperative 5-ALA fluorescence is ineffective at guiding LGGs because they do not produce a level of fluorescence that is visible to the naked eye (38). Hence, the approaches of intraoperative confocal microscopy have been developed and used to visualize 5-ALA-based tumor fluorescence in LGGs when exposed to resection procedures. With this microscopy, PpIX fluorescence has been detected in cellular infiltration identified at the tumor margins of WHO grade I and II gliomas (61).

## Intraoperative stimulation mapping and awake craniotomy approach

Because it allows for a more precise identification of functional areas (especially in the dominant hemisphere), intraoperative stimulation (IS) mapping has emerged as the standard treatment of choice for eloquent tumors. This allows surgeons to achieve higher extents of resection (EOR) and reduce postoperative morbidity.

This technique involves electrical stimulation to depolarize a focal area of the functional cortex. An electrode precisely stimulates the focal neurons to depolarize, passes the signal within the area of interest, and causes local excitation, inhibition, or perhaps diffusion to distant areas (40). The whole procedure can be monitored by an electrophysiologist if the patient is performing a motor or language task. With the help of a bipolar probe, the neurosurgeon can perform more precise mapping.

Sometimes the nidus of gliomas is located within functional cortical areas of the brain. Neurosurgeons cannot use the classic anatomy of the central nervous system (CNS) to predict functional areas (such as language or motor sites) because of individual cortical heterogeneity (62, 63). The mass effects of gliomas can distort the topography of the brain, and the brain’s plasticity can cause functional networks to be rearranged (64).

A meta-analysis of IoSM enrolled patients diagnosed with supratentorial gliomas; the result showed that the gross total resections were 75% of the patients whose resection was guided by IoSM, compared with 58% of the patients without intraoperative mapping; the IoSM did not influence the extent of the resection (65). Another meta-analysis shared similar results, especially the outcome of delayed severe deficits, which demonstrated a lower incidence (3.4%) in patients with IoSM significantly (63), and the deficits were always transient because of brain structures adjacent to the resection cavity. The use of IoSM during resection surgery reduces late, severe neurological deficits (65). The recommendation for awake craniotomy (AC) guided surgery is for gliomas affecting the dominant mid-to-posterior frontal, temporal, and mid-to-anterior parietal lobes. AC techniques can monitor more complex cognitive functions such as spatial and emotional recognition more effectively (41).

Based on some experience, the maximum duration that patients can stay conscious and complete mapping tasks is about 1 h. During this period there are several task series for IoSM, such as language tasks (the major tasks are number counting and picture naming) and non-language tasks (vision and other higher cognitive functions include calculation, working memory, and music) (66, 67). When tumors are near language or motor networks, it is recommended that direct cortical stimulation be used to help preserve function in appropriately selected patients. This often involves proceeding with an awake craniotomy, for which technical nuances and anesthesia considerations have been previously reported (68). If the gliomas are located within the area associated with severe complications, the rate of permanent complications will be about 10% with the use of AC mapping techniques (69).

A random-effects meta-analysis showed that AC (90.1%) had a higher mean EOR than general anesthesia (GA) (81.7%), which was found to be the case (p = 0.06). For analysis, neurological deficits were divided according to their severity and timing. Early neurological deficits, late neurological deficits, and non-severe and severe morbidity were not significantly different between patients who underwent AC and GA, respectively. The results suggested that IoSM when resecting gliomas located in the eloquent area can be carried out safely and effectively with AC (70).

## Other adjunct IoSM methods

There are some other adjunct IoSM methods. Functional MRI (fMRI) mainly provides vital information regarding the location of sensory and motor pathways, but it is unable to map language sites (71). Somatosensory-evoked potential (SSEP) phase-reversal techniques can be used to identify the location of the primary sensory cortex. However, the level of SSEP is only useful for locating the primary somatosensory cortex (72).

## Augmented reality high-definition fiber tractography and fluorescein

As with simulating three-dimensional virtual objects with real objects, the application of augmented reality (AR) in neurosurgery has the potential to change the way neurosurgeons plan and perform surgical procedures. The AR neuronavigation system has been used in surgical planning (73), integrating MR or CT images into the surgical field (74). López et al., in a review, illustrated that as the second most frequent use in brain tumors, AR enabled the neurosurgeon to locate the fiber tracts and guide resection (42). It can improve intraoperative safety. It facilitates and simplifies the selective anatomy of the lesion and adjacent structures, although there is no established method for precise measurement of 3D error and bias in deep injuries.

DTI high-definition fiber tractography (HDFT) has been tested as a useful tool for glioma resection planning and assessment of postoperative connectivity of the fiber tracts (43). Sodium fluorescein (F) has been used as a fluorescent dye in HGGs to increase the EOR (75). Although there is still a wide range of limitations, there are several studies about the combined utility of AR and HDFT-F. AR HDFT-F improves the neurosurgeon’s intraoperative spatial location and allows for differential visualization of each tract as needed. Luzzi (1) enrolled 117 patients newly diagnosed as supratentorial HGGs, of whom 54 underwent surgery with the AR HDFT-F technique and 63 with conventional neuronavigation surgery. The results suggested that the AR HDFT-F group had a higher extent of resection and longer progression-free survival, although there was no significant difference in complication rates between the two groups. Surgery with AR HDFT-F is regarded as a safe and effective procedure for patients’ neurofunction recovery. However, the present procedures involve only GA patients, and the whole process is still limited by the DTI’s only supply of anatomical information (76).

There are some *other intraoperative tools* that have been described to achieve maximal tumor resection, and most of them are still in debate.

(1) Intraoperative hand-held microscopy

This is a technique in which a single optical fiber combined with miniaturized scanning and optical systems supplies high-resolution images (77). It can solve problems at the cellular level. The histological characteristics of gliomas, meningiomas, and central neurocytomas have been distinguished by intraoperative confocal microscopy to visualize fluorescein (78). Further study is needed to determine whether this technique can be used to complement resection procedures and conventional neuropathological diagnostic techniques.

(2) Intraoperative mutational analysis

The analysis can be used in real time to theoretically determine the true tumor margins. All these methods include genotyping for known tumor mutations using PCR-based approaches, such as isocitrate dehydrogenase 1 or 2 (IDH1/2) aberrations (79); mass spectroscopy based on defined tumor spectral profiles, which is similar to magnetic resonance spectroscopy techniques (80); All these methods need to be further tested for specificity and sensitivity in resectioning gliomas.

## Conclusions

Preoperative (such as neuronavigation systems and DTI tractography) and intraoperative (including IoMRI, IoUS, fluorescence-guided surgery, IoSM and AC approaches, AR HDFT-F, intraoperative hand-held microscopy, and mutational analysis) surgical options for gliomas therapy supply a lot of choices for neurosurgeons to improve the greater extent of resections of any grade of gliomas (**Table 1**). The neuronavigation system is always used for preoperatively precise planning. DTI tractography can plan surgical fibro-parcel pathways, keeping away from significant white-matter packages. IoMRI can solve the intraoperative brain shift. IoUS can be easily incorporated into operations to detect residuals above 1 cm in diameter. Fluorescence-guided surgery can detect the tumors in HGGs and LGGs with confocal microscopy and fluorescence. IoSM and AC approaches can be used for gliomas located in the eloquent area. AR HDFT-F can improve intraoperative spatial location and allow for differential visualization of each tract. As the methods need to be further tested, intraoperative hand-held microscopy and mutational analysis also provide the methods for glioma resection. The development of surgical techniques has changed the principles of gliomas and characterized them as accurate, effective, and real-time to maximize tumor resection, preserve neurological function of the eloquent area near the lesion, decrease morbidity, and improve outcomes.

**Table 1:** Perioperative treatment options.

Timepoint	Techniques	Advantages
Preoperative	Neuronavigation systems	(1) preoperative precise planning of the craniotomy in real time and the identification of small intracranial lesions. (2) can add functional MRI and DTI tractography information intraoperatively.
Preoperative	DTI tractography	Plan major fibro-parcel pathways in 3D and keeping away from significant white-matter packages.
Intraoperative	Intraoperative MRI (IoMRI)	Solve the problem of the brain shift and increase the rate of EOR.
Intraoperative	Intraoperative ultrasound (IoUS)	The tool that can be easily incorporated into operative workflow to detect the residual above 1cm in diameter.
Intraoperative	Fluorescence-guided surgery	(1) fluorescence of red-violet light: for the detection of tumor tissues during HGGs resection. (2) intraoperative confocal microscopy with fluorescence: for LGGs.
Intraoperative	Intraoperative Stimulation Mapping (IoSM) and Awake craniotomy (AC) approach	Resect gliomas located in the eloquent area can be carried out safely and effectively.
Intraoperative	Augmented reality high-definition fiber tractography and fluorescein (AR HDFT-F)	Improve intraoperative spatial location and allow for differential visualization of each tract.
Intraoperative	Intraoperative hand-held microscopy	Solve problems at cellular resolution with a single optical fiber.
Intraoperative	Intraoperative mutational analysis	Genotyping and mass spectroscopy in real time to determine the true tumor margins.

## Author contributions

XC is the corresponding author, and he designed the content. ZY works on the analysis selected data and wrote the manuscript. CZ, SZ, JP, and YZ are responsible for collecting the papers focused on the surgical treatment of the gliomas from hundreds of papers. All authors contributed to the article and approved the submitted version.
